# The lipid phosphatase INPP4B controls pancreatic cancer cell migration and invasion by regulating fibronectin exocytosis

**DOI:** 10.1016/j.jbc.2025.110716

**Published:** 2025-09-15

**Authors:** Golam T. Saffi, Nicholas Kleine, Leonardo Salmena

**Affiliations:** Department of Pharmacology and Toxicology, University of Toronto, Toronto, Ontario, Canada

**Keywords:** inositol polyphosphate 4-phosphatase type II, transient receptor potential cation channel mucolipin subfamily member 1, pancreatic ductal adenocarcinoma, fibronectin 1, focal adhesion kinase, phosphoinositide kinase, FYVE-type zinc finger containing, phosphatidylinositol-3,4-bisphosphate, phosphatidylinositol-3-monophosphate, phosphatidylinositol-3,5-bisphosphate, extracellular matrix, Clinical Proteome Tumour Analysis Consortium, gene expression profiling interactive analysis, The Cancer Genome Atlas-Pancreatic Ductal Adenocarcinoma study, Gene Expression Omnibus

## Abstract

Increased expression of inositol polyphosphate 4-phosphatase, type II (INPP4B) correlates with aggressive phenotypes in pancreatic ductal adenocarcinoma (PDAC). Although prior studies have linked INPP4B to lysosome positioning, exocytosis, and enhanced cell migration and invasion in PDAC, the specific mechanisms underlying these processes remain unclear. In this study, we demonstrate that INPP4B promotes fibronectin 1 (FN1) secretion *via* transient receptor potential cation channel mucolipin subfamily member 1–dependent lysosomal exocytosis. In addition, we show that INPP4B-mediated regulation of F-actin formation, focal adhesion kinase activation, and increased cell migration and invasion depend on FN1 exocytosis. These findings underscore the INPP4B–transient receptor potential cation channel mucolipin subfamily member 1–FN1 axis as a critical contributor to PDAC aggressiveness and identify it as a promising candidate for the development of new therapeutic strategies.

Lysosomes are crucial in driving the aggressiveness of pancreatic ductal adenocarcinoma (PDAC), impacting tumor growth, metastasis, and drug resistance (1, 2). Beyond traditional degradative roles, lysosomes act as signaling hubs, regulating processes such as nutrient signaling, immune response, and plasma membrane repair (1, 3, 4, 5, 6, 7, 8). This study investigated the influence of lysosome functions and dynamics on PDAC progression.

Inositol polyphosphate 4-phosphatase, type II (INPP4B) is a tumor-promoting lipid phosphatase and biomarker of poor prognosis in PDAC (9, 10). INPP4B has been shown to regulate lysosomal biogenesis, reformation, peripheral lysosome redistribution, and lysosomal exocytosis by modifying the phosphoinositide repertoire on lysosome membranes (11, 12, 13, 14, 15). In PDAC cells, overexpression (OE) of INPP4B led to elevated levels of phosphatidylinositol-3,5-bisphosphate on lysosomal membranes, which led to transient receptor potential cation channel mucolipin subfamily member 1 (TRPML-1) activation, Ca^2+^ release, lysosome exocytosis, and subsequent cytoskeletal organization, resulting in increased cell migration and invasion (13, 16, 17, 18, 19, 20). Despite a key role for INPP4B in the regulation of lysosomes and PDAC cell motility, the lysosomal factors that mediate these processes upon exocytosis remain to be determined.

Fibronectin 1 (FN1) is an extracellular matrix protein released by fibroblasts and tumor cells, serving as a major component of the PDAC tumor microenvironment that supports adhesion, migration, proliferation, and cell differentiation (21, 22). Through binding to integrins, FN1 can trigger F-actin reorganization and stimulate cell migration (23, 24, 25, 26). Importantly, FN1 can be trafficked to lysosomes *via* caveolin-dependent endocytosis (22, 25, 27, 28). This study provides evidence that INPP4B promotes PDAC cell migration and invasion through a mechanism involving the exocytosis of lysosome-resident FN1.

## Results and discussion

### FN1 is required for INPP4B-mediated F-actin formation in PDAC cells

**Figure 1:**
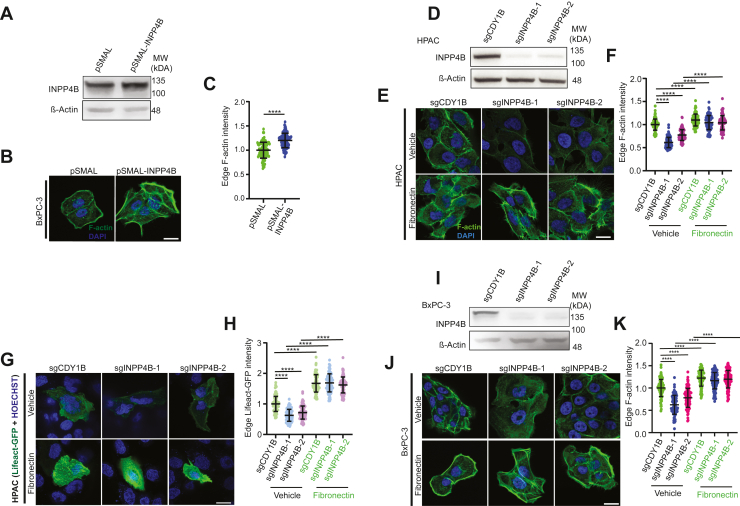
**INPP4B and FN1 interact to regulate the actin cytoskeleton.***A,* immunoblot demonstrating OE of *INPP4B* in BxPC-3. *B* and *C,* fluorescence imaging and quantitation of edge-F-actin in *INPP4B*-overexpressing BxPC-3 cells costained with Alexa^488^-phalloidin and DAPI. *D,* immunoblot of *INPP4B* KO HPAC cells. *INPP4B* KO HPAC cells treated with 20 μg/ml FN1 or vehicle assessed through F-actin IF with phalloidin staining (*E* and *F*) or (*G* and *H*) Lifeact-GFP and Hoescht 33342. *I,* immunoblot demonstrating *INPP4B* KO BxPC-3 cells. *J* and *K,* fluorescence imaging and quantitation of edge-F-actin in *INPP4B* KO BxPC-3 cells treated with 20 μg/ml FN1 or vehicle costained with Alexa^488^-phalloidin and DAPI. The scale bar represents 25 μm. Data represent ±SD from 90 to 100 cells assessed from three independent experiments per treatment condition. DAPI, 4′,6-diamidino-2-phenylindole; FN1, fibronectin 1; IF, immunofluorescence; INPP4B, inositol polyphosphate 4-phosphatase, type II; OE, overexpression.

Given the critical role of FN1 in promoting F-actin assembly (23, 29, 30, 31, 32), we hypothesized that this process may be controlled by INPP4B. In support of this, *INPP4B* OE led to significantly increased F-actin assembly at the cell periphery (Fig. 1, *A*–*C*), In *INPP4B* KO cells, F-actin intensity was significantly reduced (Fig. 1, *D*–*K*). This effect was reversed after treatment with FN1 in HPAC (Fig. 1, *E*–*H*) and BxPC-3 cells (Fig. 1, *J* and *K*). These results provide evidence that INPP4B-mediated F-actin assembly is dependent on FN1.

### INPP4B expression affects focal adhesion kinase activation *via* FN1 in PDAC cells

**Figure 2:**
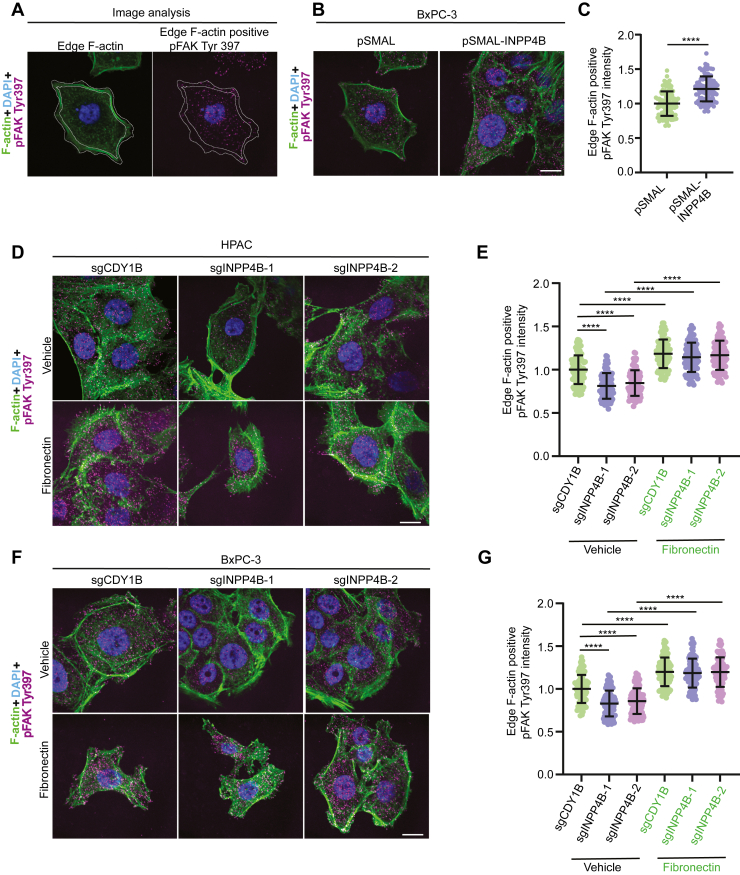
**FAK activation at the cell periphery is regulated by INPP4B in an FN1-dependent manner.***A,* to quantitate FAK activation, we manually drew a region of interest around phalloidin-stained cell edge F-actin, and this region was replicated to identify and quantitate cell edge phospho-FAK Tyr397 region. F-actin intensity was normalized across treatments to identify differences in phospho-FAK Tyr397 intensity without variations in F-actin intensity. *B*, representative micrographs and (*C*) quantitation of FAK activation in BxPC-3 cells transduced with *pSMAL* or *pSMAL-INPP4B* and probed for F-actin with phalloidin (*green*), DAPI (*blue*) for nucleus, and immunostained with phospho-FAK Tyr397 (*magenta*). Representative micrographs and quantitation of FAK activation in *INPP4B* KO HPAC *(D*, *E*) and *INPP4B* KO BxPC-3 (*F* and *G*) cells treated with vehicle or 20 μg/ml FN1. The scale bar represents 20 μm. Data represent ±SD from 90 to 100 cells assessed from three independent experiments per treatment condition. DAPI, 4′,6-diamidino-2-phenylindole; FAK, focal adhesion kinase; FN1, fibronectin 1; INPP4B, inositol polyphosphate 4-phosphatase, type II.

Focal adhesion kinase (FAK) is activated downstream of FN1 signaling to promote cell motility (33, 34, 35, 36, 37, 38). To determine whether INPP4B could also activate FAK, we measured the levels of phospho-FAK^Tyr397^ at F-actin–rich cell edges using immunofluorescence (Fig. 2*A*). Phospho-FAK^Tyr397^ was increased upon *INPP4B* OE (Fig. 2, *B* and *C*) and decreased upon *INPP4B* KO (Fig. 2, *D*–*G*). The FN1 dependence of this effect was demonstrated by restoration of phospho-FAK^Tyr397^ levels in *INPP4B* KO cells upon FN1 treatment (Fig. 2, *D*–*G*). These data support the existence of a specific INPP4B–FN1 axis driving FAK activation.

### FN1 is localized within lysosomes, which are distributed in an INPP4B-dependent manner

To investigate whether INPP4B influences FN1 localization, we aimed to replicate data showing that FN1 is trafficked within lysosomes (22, 25, 27, 28). Consistent with these reports, we observed enhanced colocalization of FN1 in lysosome-associated membrane protein 1 (LAMP1)–positive structures, as compared with Rab5-positive structures, suggesting specific lysosomal localization (Fig. S1, *A* and *B*). Following treatment of PDAC cells with the PIKfyve (phosphoinositide kinase, FYVE-type zinc finger containing) inhibitor apilimod, FN1 accumulated within approximately 70% of LAMP1-positive enlarged lysosomes, further supporting its presence within the lysosomal lumen (Fig. S1, *C* and *D*). Treatment of PDAC cells with bafilomycin A1, a v-ATPase inhibitor, increased total cell and LAMP1-colocalized FN1 intensity by blocking lysosomal acidification and protein degradation (2, 39, 40, 41), (Fig. S1, *E*–*G*). Together, these results provide evidence that FN1 is localized and degraded in PDAC cell lysosomes.

**Figure 3:**
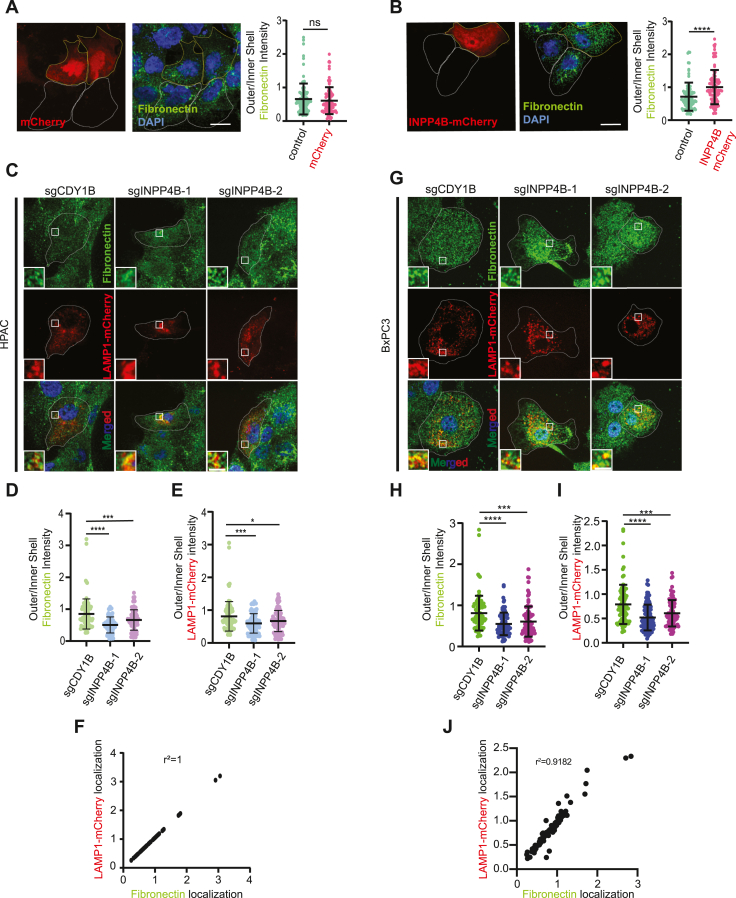
**Intracellular FN1 localization is regulated by lysosomes.** HPAC cells transfected with (*A*) pmCherry or (*B*) pmCherry-INPP4B were immunostained for FN1 (*green*) and assessed for outer/inner shell FN1 intensity. Representative micrographs and quantitation of outer/inner shell LAMP1 and FN1 intensity in *INPP4B* KO HPAC (*C–E*) and *INPP4B* KO BxPC-3 (*G–I*) cells transiently expressing LAMP1-mCherry and immunostained for FN1 (*green*). Linear regression and correlation between LAMP1-mCherry localization and FN1 localization in HPAC (*F*) or BxPC-3 (*J*) cells. The scale bar represents 15 μm (*A* and *B*), 5 μm (*C* and *G*). Data represent ±SD from 90 to 100 cells assessed from three independent experiments per treatment condition. FN1, fibronectin 1; INPP4B, inositol polyphosphate 4-phosphatase, type II; LAMP1, lysosome-associated membrane protein 1.

We hypothesized that INPP4B affects lysosomal FN1 cargo localization, given its role in lysosomal positioning (13). To test this, we analyzed how altering INPP4B expression impacts FN1 distribution by measuring lysosome and FN1 fluorescence in perinuclear and juxtamembrane regions of PDAC cells (13). OE of *INPP4B-mCherry* led to the redistribution of both FN1 and LAMP1 toward the cell periphery, which was not observed with the *mCherry* control construct (Fig. 3, *A* and *B*). Similar results were achieved in BxPC-3 cells with *INPP4B* OE (Fig. S1, *H*–*J*). *INPP4B* KO resulted in a shift of both LAMP1-mCherry and FN1 toward the perinuclear region (Fig. 3, *C*–*E*, *G*–*I*). Notably, FN1 and LAMP1 displayed nearly identical localization patterns (Fig. 3, *F* and *J*). These findings provide evidence that FN1 is localized within lysosomes and that INPP4B plays a key role in their dynamic cellular redistribution.

### FN1 secretion is dependent on INPP4B and TRPML-1-mediated lysosomal exocytosis

**Figure 4:**
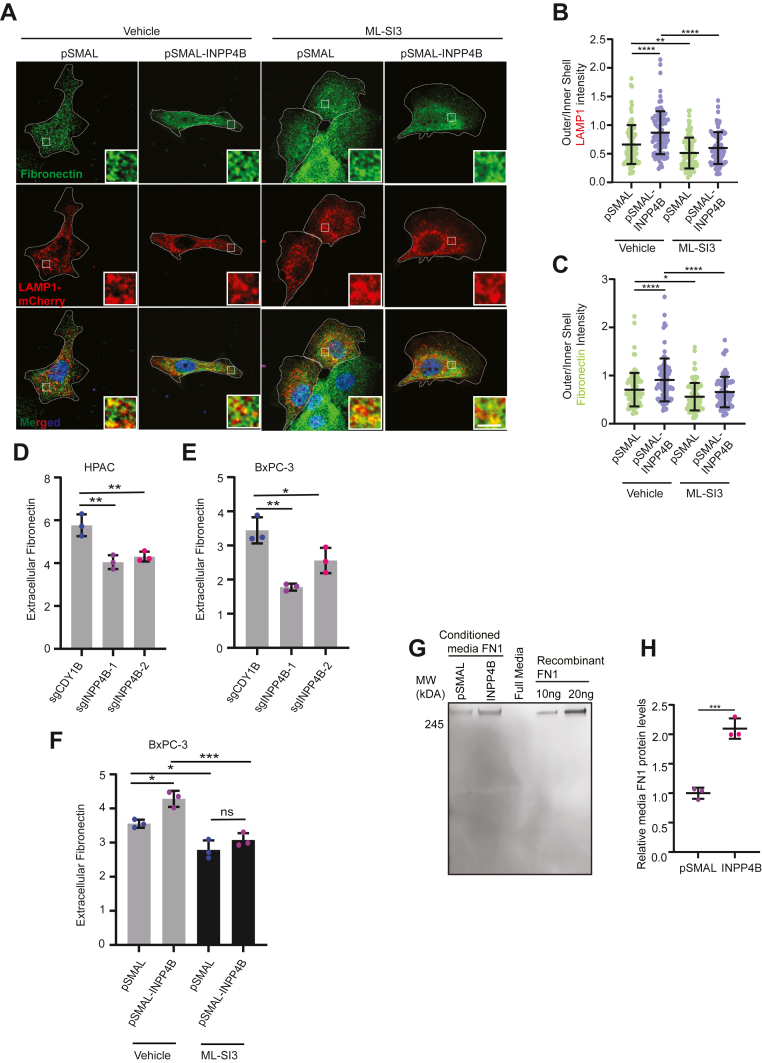
**The INPP4B–TRPML-1 axis can mediate lysosomal exocytosis and influence FN1 secretion.***A,* representative micrographs and (*B* and *C*) quantitation of outer/inner shell LAMP1 and FN1 intensity in BxPC-3 cells transduced with *pSMAL* or *pSMAL-INPP4B* and transiently expressing LAMP1-mCherry (*red*) and immunostained for endogenous FN1 (*green*). Cells were treated with either vehicle or ML-SI3. The scale bar represents 5 μm. *INPP4B* KO HPAC (*D*) and *INPP4B* KO BxPC-3 (*E*) cells were assessed for extracellular FN1 levels after 24 h of incubation. *F,* BxPC-3 cells transduced with *pSMAL* or *pSMAL-INPP4B* and treated with vehicle or ML-SI3 for 24 h followed by extracellular FN1 measurement. *G* and *H,* BxPC-3 cells transduced with *pSMAL* or *pSMAL-INPP4B* incubated in 0.1% FBS containing DMEM media for 24 h followed by collection of conditioned media and FN1 immunoblotting compared with recombinant FN1. Data represent 90 to 100 cells assessed from three independent experiments per treatment condition (*A–C*) or three independent experiments per treatment condition (*D–H*)*.* DMEM, Dulbecco's modified Eagle's medium; FBS, fetal bovine serum; FN1, fibronectin 1; INPP4B, inositol polyphosphate 4-phosphatase, type II; LAMP1, lysosome-associated membrane protein 1; TRPML-1, transient receptor potential cation channel mucolipin subfamily member 1.

Our previous work showed that INPP4B OE stimulates lysosomal exocytosis *via* TRPML-1-dependent calcium release (13). Building upon these findings, we hypothesized that INPP4B–TRPML-1 signaling also regulates the relocalization and exocytosis of FN1. To test this, *INPP4B*-OE cells were treated with ML-SI3, a specific inhibitor of TRPML-1 (42). ML-SI3 antagonized INPP4B-induced peripheral localization of both LAMP-1 (Fig. 4, *A* and *B*) and FN1 (Fig. 4, *A* and *C*), suggesting that FN1 localization is regulated by lysosomal positioning through the INPP4B–TRPML-1 signaling axis.

To test if INPP4B promotes FN1 secretion *via* lysosomal exocytosis, we measured FN1 levels in conditioned media (CM) by ELISA. FN1 levels were significantly reduced in *INPP4B* KO cells (Fig. 4, *D* and *E*) and significantly increased in *INPP4B*-OE cells (Fig. 4*F*). Crucially, ML-SI3 reduced FN1 levels in CM, providing evidence that FN1 secretion is dependent on TRPML-1-mediated lysosomal exocytosis (Fig. 4*F*). Western blot of CM using an anti-human FN1 antibody provided additional evidence that FN1 levels were higher in *INPP4B-*OE cells (Fig. 4, *G* and *H*). No bovine FN1 was detected by Western blot (Fig. 4, *G* and *H*). Importantly, Western blots also demonstrate that the extracellular FN1 was almost exclusively of full length (Fig. 4*G*). Collectively, these results indicate that INPP4B–TRPML-1 signaling controls lysosomal exocytosis of FN1 into the extracellular matrix.

### INPP4B regulation of PDAC migration is FN1 dependent

**Figure 5:**
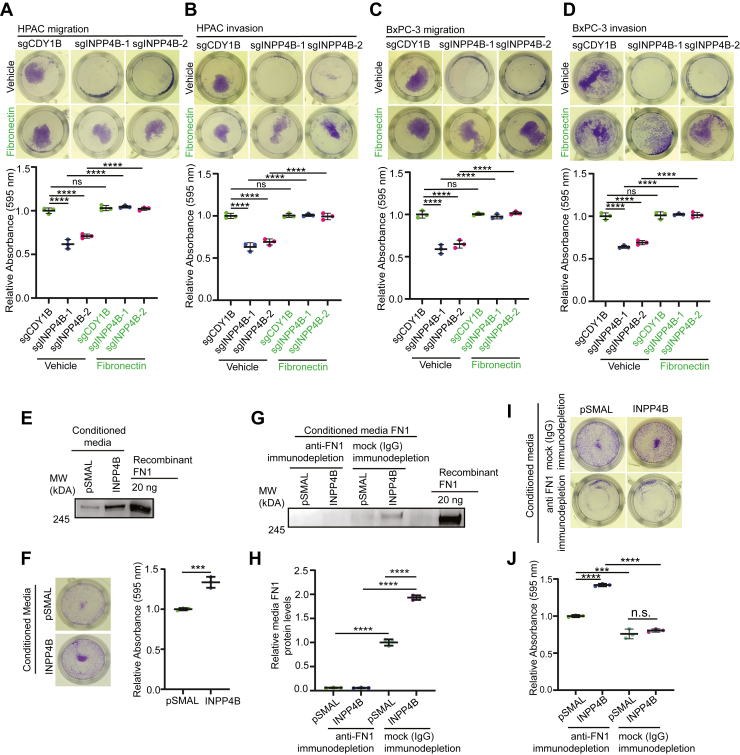
**INPP4B-mediated migration and invasion are dependent on FN1.***A–D,* representative images and quantitation of *INPP4B* KO in HPAC or BxPC-3 with vehicle or 2 μg/ml FN1, assessed for transwell migration or transwell invasion. *E* and *F,* conditioned media collected from BxPC-3 cells transduced with *pSMAL* or *pSMAL-INPP4B* and applied to parental BxPC-3 cells for transwell migration. *G–J,* conditioned media collected from BxPC-3 cells transduced with *pSMAL* or *pSMAL-INPP4B*, immunodepleted with anti-FN or mock IgG antibody and applied to parental BxPC-3 cells for transwell migration. Data represent at least three independent experiments per treatment condition. FN1, fibronectin 1; IgG, immunoglobulin G; INPP4B, inositol polyphosphate 4-phosphatase, type II.

We next tested if INPP4B OE promotes PDAC cell movement *via* FN1. *INPP4B* KO cells showed reduced wound healing, which was fully restored by exogenous FN1. (Fig. S2, *A*–*C*). Next, in transwell migration and invasion assays, *INPP4B* KO exhibited significantly reduced migratory and invasive capabilities (Fig. 5, *A*–*D*). Addition of exogenous FN1 restored the migratory and invasive capacities of *INPP4B*-KO cells (Fig. 5, *A*–*D*). These results support a role for FN1 in INPP4B-mediated cell migration and invasion.

Next, to evaluate the specific role of FN1 in INPP4B-driven cell migration, we performed migration assays using parental BxPC3 cells using CM from both *INPP4B*-OE and control BxPC3 cells. When applied in migration assays, CM from *INPP4B*-OE BxPC3 cells promoted the migration of parental BxPC3 cells compared with media from controls (Fig. 5, *E* and *F*). To specifically assess the role of FN1, we performed immunodepletion with anti-FN1 antibodies or control rabbit immunoglobulin G (IgG) (Fig. 5, *G* and *H*). Immunodepletion of FN1 from both *INPP4B*-OE and control CM significantly reduced promigratory potential (Fig. 5, *I* and *J*), suggesting that FN1 is a key contributor to INPP4B-induced migration.

### INPP4B expression in PDAC is associated with genes involved in cell migration

Analysis of PDAC patient databases revealed that FN1 is frequently overexpressed in PDAC compared with normal pancreatic tissue (Fig. S3, *A*–*C*). Elevated FN1 levels correlate with poor patient survival (Fig. S3, *D* and *E*), supporting a potential oncogenic role for FN1 in PDAC. Interestingly, The Cancer Genome Atlas-Pancreatic Ductal Adenocarcinoma study dataset showed a strong correlation between *FN1* and *INPP4B* expression (Fig. S3*F*), suggesting potential coregulation of these genes in PDAC. However, experiments to validate this correlation in HPAC and BxPC-3 cells yielded inconsistent and inconclusive results (Fig. S3, *G*–*I*). Therefore, further investigation is needed to determine if a causal link exists between FN1 and INPP4B expression.

Expression levels of other cell surface markers involved in migratory responses, such as E-cadherin and β1-integrin (17) were observed to be elevated or decreased upon *INPP4B* OE and KO, respectively (Fig. S4, *A*–*F*). Notably, their levels were unaffected by ML-SI3 or FN1, indicating regulation independent of lysosomal exocytosis or FN1 secretion. By examining The Cancer Genome Atlas-Pancreatic Ductal Adenocarcinoma study dataset, we identified strong positive correlations between *INPP4B* and both *ITGB1* and *CDH1* (Fig. S4, *G* and *H*). TaqMan Real-Time PCR analysis of *ITGB1* and *CDH1* expression in *INPP4B* OE and KO cells confirmed these correlations (Fig. S4, *I* and *J*). These results demonstrate that INPP4B promotes transcriptional upregulation of E-cadherin and β1-integrin independent of FN1, revealing an additional mechanism by which INPP4B may promote migratory responses.

While the link between INPP4B, lysosome positioning, exocytosis, and cell migration/invasion has been established, the underlying mechanisms remain unclear. This study reveals that FN1, released during INPP4B-mediated lysosomal exocytosis, leads to the regulation of the actin cytoskeleton, as shown by activation of F-actin assembly and FAK phosphorylation. This suggests a novel mechanism by which INPP4B, through its regulation of TRPML-1 and lysosomal exocytosis resulting in subsequent FN1 release, drives cell motility and invasion in the PDAC tumor microenvironment.

In our model, elevated INPP4B activates TRPML-1, thereby promoting lysosomal exocytosis and FN1 release. Exocytosed FN1 enhances F-actin assembly and FAK activation, both critical for cell motility and invasion. Thus, the INPP4B–FN1 axis emerges as a potential therapeutic target, where inhibiting INPP4B or blocking FN1 secretion may suppress PDAC cell migration and invasion, limiting disease progression.

## Experimental procedures

### Cell lines

BxPC-3 (from a 61-year-old female with PDAC), HPAC (from a 64-day-old female with PDAC), and human embryonic kidney 293T cells were obtained from the American Type Culture Collection. They were cultured at 37 °C with 5% CO_2_ in Dulbecco's modified Eagle's medium (DMEM) supplemented with 10% fetal bovine serum (FBS) and 1% penicillin–streptomycin. Cell lines were authenticated *via* short tandem repeat analysis (The Centre for Applied Genomics, SickKids), routinely passaged with plasmocin, and confirmed to be mycoplasma free using the PlasmoTest *Mycoplasma* Detection Kit.

### Plasmids

*GFP* was replaced with *puromycin*, and a codon-optimized human *3xFLAG-INPP4B* sequence was cloned into *pSMAL* (a gift from John Dick and Peter Van Galen (https://www.addgene.org/161785/) to generate *pSMAL-puro and pSMAL-INPP4B-puro*. Control sgRNA targeting the nonessential gene *sgCDY1B* (TCTGCACCAGGACGTGACAA), *sgINPP4B-1* (ATACTCCAGCACCGAAATTG), and *sgINPP4B-2* (GATGTACAGGGACAAAAGGT) were cloned into *LentiCRISPR-V2,* a gift from Feng Zhang (https://www.addgene.org/52961/). *pmCherry-INPP4B* was derived from *mCherry-FKBP-INPP4B*, a gift from Gerry Hammond (https://www.addgene.org/116864/). The *mCherry-Lysosomes-20* (*mCherry-LAMP1*) was a gift from Michael Davidson (https://www.addgene.org/55073/). *Rab5-mRFP* was a gift from Ari Helenius (https://www.addgene.org/14437/). *Lifeact-EGFP,* a reporter to detect cytoplasmic actin filaments, was a kind gift from Dyche Mullins (https://www.addgene.org/58470/).

### Lentivirus production and infection

Lentiviral packaging plasmids *psPAX2* (a gift from Didier Trono https://www.addgene.org/12260/) and *VSVG* (a gift from Tannishtha Reya https://www.addgene.org/14888/) were used to generate lentiviral particles as before (13).

### Transient gene transfection

Transient gene transfections were performed using Fugene HD (Promega) according to the manufacturer's instructions, employing a DNA:Fugene ratio of 3:1 for 48 h. This was followed by a 1× PBS wash and supplementation with complete DMEM.

### Drug treatment of cell lines

Soluble FN1 (R&D Systems) was applied to HPAC and BxPC-3 cells for 24 h at the indicated doses. ML-SI3 (Selleckchem) was used at 10 μM for 24 h. Apilimod (Selleckchem) was used at 500 nM for 1 h. Bafilomycin (Selleckchem) was used at 15 nM for 24 h.

### Quantitative RT–PCR

RNA was extracted using the RNeasy mini kit (Qiagen) and reverse transcribed using the Superscript IV Vilo cDNA synthesis kit (Thermo). Complementary DNA was amplified using TaqMan Fast Advanced Master Mix (Thermo) and TaqMan Assays with QuantStudio 3 Real-Time PCR system (Thermo). TaqMan assays include *FN1* (Hs01549976_m1), *ITGB1* (Hs01127536_m1), *CDH1* (Hs01023895_m1), *ACTB* (Hs01060665_g1), and *GAPDH* (Hs02786624_g1). Gene expression was determined using the relative quantification (ΔΔCt method) and normalization to GAPDH.

### Immunoblotting

Whole-cell lysates were generated using 1× radioimmunoprecipitation assay buffer supplemented with protease inhibitor cocktail and immunoblotted with antibodies against INPP4B (1:1000 dilution; Cell Signaling Technologies, #14543), fibronectin (1:500 dilution; Cell Signaling Technologies, #26836 human specific, no crossreactivity to bovine FN1), or β-Actin (1:1000 dilution; Cell Signaling Technologies, #4967). Blots were visualized using KwikQuant Digital Western Blot Detection System (Kindle Biosciences). Protein quantification was performed using ImageJ (ImageJ; Washington, DC). Pixel values inverted to correlate protein levels with pixel intensity, followed by quantification of pixel intensity of individual protein bands.

### Immunofluorescence

For immunostaining, cells were fixed with 4% paraformaldehyde for 15 min, permeabilized with 0.05% Triton X-100 or 20 μM digitonin, and blocked with 3% bovine serum albumin in PBS. They were then incubated with specific primary antibodies (anti-FN1, phospho-FAK, CD29, or E-cadherin) and respective DyLight-labeled secondary antibodies. F-actin was stained with Alexa Fluor 488 Phalloidin and 4′,6-diamidino-2-phenylindole for 10 min.

### Microscopy imaging

For imaging applications, fluorescence spinning disc confocal microscopy was used with an Olympus IX81 inverted microscope connected to a Hamamatsu C900-13 EMCCD camera with 60× 1.35 numerical aperture objective that was controlled by the program software Volocity 6.3.0 (Quorum Technologies, Inc). Live imaging was performed at 37 °C and 5% CO_2_ in a chamber with complete DMEM. The microscope used was equipped with standard filters for all the fluorophores used in the study.

### Quantitation of extracellular FN1

To measure extracellular secreted FN1 levels within the media, phenol red–free DMEM (Thermo Fisher) supplemented with 0.1% FBS was collected at 0 and 24 h. Total extracellular FN1 was measured according to the manufacturer's instructions (Thermo Fisher) using a colorimetric sandwich ELISA. Total FN1 measurements normalized for 24 h relative to 0 h.

### Transwell migration and invasion assays

Approximately 1.2 × 10^5^ cells were resuspended in DMEM with 0.1% FBS in the upper chambers of 12-well ThinCert inserts (8 μm) and allowed to migrate for 24 h toward 10% FBS in the lower chamber. Cells on the underside were fixed, stained with crystal violet, washed, dried, and photographed. For invasion assays, the membrane was coated with Matrigel, polymerized at 37 °C for 3 h, then seeded with 5.5 × 10^5^ cells. A 2 μg/ml FN1 dose was used, which had no effect on baseline migration or invasion.

### Collection and immunodepletion of FN1 from CM

BxPC-3 cells were cultured at 5 × 10^5^ per well in 6-well plates in DMEM with 0.1% FBS for 24 h. CM was precleared with Protein A/G magnetic beads for 2 h and then incubated overnight with beads conjugated to anti-FN1 or IgG control. Beads were removed, and the media underwent two additional 24-h antibody incubations. The FN1-depleted or IgG-treated media were then used for transwell migration assays or heat denatured for immunoblotting.

### Statistical analyses

All experiments were repeated a minimum of three times. Data points were plotted in GraphPad Prism. One-way ANOVA’s Tukey’s *post hoc* test was performed for statistical significance for comparison between more than two treatment conditions or unpaired two-tailed parametric *t* test for comparison between two treatment conditions. ∗*p* < 0.05, ∗∗*p* < 0.01, ∗∗∗*p* < 0.001, and ∗∗∗∗*p* < 0.0001. *p* Values <0.05 were considered statistically significant.

## Data availability

All data and reagents presented in this study are available upon request.

## Supporting information

This article includes supporting information that provides additional figures and detailed experimental procedures (7, 12, 43, 44, 45, 46).

## Conflict of interest

The authors declare that they have no conflicts of interest with the contents of this article.

## Supporting information

Supplementary Figure 4

Supplemental Figure Raw Western Blot images
