# Supplementary material for: The lipid phosphatase INPP4B controls pancreatic cancer cell migration and invasion by regulating fibronectin exocytosis
Source: J Biol Chem. 2025 Sep 15;301(10):110716. doi: 10.1016/j.jbc.2025.110716 (PMC12547244; doi:10.1016/j.jbc.2025.110716)
Supplement: Supplemental Information [file mmc1.pdf]

Saffi et al. Supplemental Information

Supplemental Materials and Methods

Flow cytometry

To assess cell surface E-cadherin and β1 Integrin levels, HPAC and BxPC-3 cells trypsinized and incubated with 1:100 dilution of PE anti-human CD29 (β1 Integrin) clone TS2/16 or FITC anti-human CD324 (E cadherin) clone 67A4 (BioLegend) for 15 min in dark in ice. Flow cytometry performed using Beckman Coulter Cytoflex (Beckman) measuring 10,000 events per sample.

Immunofluorescence

For immunostaining of endogenous proteins or phospho-proteins, cells were fixed for 15 min with 4% (v/v) paraformaldehyde (PFA) in PBS, permeabilized for 10 min with 0.05% (v/v) Triton X-100 in PBS and blocked with 3% (v/v) BSA in PBS. Cells were then incubated with rabbit monoclonal anti-human FN1 (Cell Signaling, #26836), or rabbit monoclonal anti-human phospho-FAK Tyr397 (Thermo Fisher, #44-625G) and donkey anti-rabbit DyLight 488 (Bethyl) antibody or goat anti-rabbit DyLight 650 (Thermo Fisher) or goat anti-mouse DyLight 650 (Thermo Fisher). For F-actin immunostaining, cells were fixed with 4% (v/v) PFA in PBS for 15 min, permeabilized for 10 min with 20 μM digitonin (Promega) and blocked with 3% (v/v) BSA in PBS. Cells were incubated with Alexa Fluor 488 conjugated Phalloidin (Thermo Fisher) and DAPI for 10 min.

Image Analysis

Distribution of immunostained FN1 or LAMP1-mCherry labelled lysosomes proceeded as previously reported, we adapted a technique previously used to draw equi-distant inner and outer shells from the nucleus followed by measuring FN1 or LAMP1 intensity ratio of outer

shell/inner shell (7, 12, 44). To quantify FN1 intensity individual cells outlined to identify regions of interest followed by measurement of mean FN1 intensity per cell. To quantify FN1 intensity over LAMP1-mCherry or Rab5-RFP positive regions within a cell, ImageJ was used to apply intensity thresholding over LAMP1-mCherry or Rab5-RFP positive regions and produce a mask. The mask was applied to the green FN1 channel to identify green channel intensity over LAMP1-mCherry or Rab5-RFP positive regions. FN1 fluorescence images for Fig. 3C were adjusted for intensity to visualise FN1 colocalization to LAMP1-mCherry particles independent of FN1 levels affected by INPP4B expression. To assess the percentage of lysosome vacuoles with internal or luminal FN1, vacuoles were first defined to be LAMP1 positive being greater than 1.5 μm in diameter as previously established (11, 45). Total number of vacuoles per cell and number of vacuoles positive for internal FN1 intensity signal per cell were counted and used to identify percentage of vacuoles positive for internal or luminal FN1. To analyse F-actin or Lifeact-GFP intensity around the cell edge, we used a previously established image analysis technique to manually draw and measure cell edge intensity for stained phalloidin or LifeAct-GFP (12). To measure phospho-FAK Tyr397, E-cadherin or β1-integrin intensity for edge F-actin positive regions of a cell, cell edge region stained with phalloidin replicated to identify edge total raw integrated pixel intensity of respective proteins within this region and divided by the total number of pixels.

Wound healing/Scratch assay

Cells were grown to full confluency in a 24-well plate and pre-treated with vehicle or FN1 for 4 hours. Two vertical scratches were created using sterile plastic tips. Cells were washed and imaged in PBS at 0 hours (time of scratch) and after 20 hours with vehicle or FN1. DIC light microscopy was used for imaging at 10x. Images were imported into ImageJ and analyzed to determine the number of pixels in the wound area at each time point.

Transwell migration and invasion assays

1.2 x 10^5 cells were resuspended in the upper chambers of 12-well ThinCert inserts, 8 µm (Greiner Bio-One) in DMEM supplemented with 0.1% FBS to ensure minimal baseline FN1 presence. DMEM supplemented with 10% FBS was added to the bottom chamber and cells were allowed to migrate for 24 h. Cells attached to the bottom of membranes were fixed with 10% formalin and stained with 0.1% crystal violet in 20% methanol, washed with PBS dried overnight and photographed. The crystal violet was dissolved in 10% acetic acid and quantified at 595 nm absorbance. PDAC cell invasion was assessed in an identical manner with the exception that the transwell membrane was coated with 1:10 dilution of Matrigel Growth Factor Reduced Basement Membrane Matrix (Corning Life Science), polymerized at 37^0C for 3 h followed by seeding with 5.5 x 10^5 PDAC cells. 2 µg/ml of FN1 -- a dose at which there was no observed change in migration or invasion in control cells -- was used for transwell assays. For transwell migration assays using CM, 0.1% FBS supplemented DMEM media was collected from BxPC-3 cells stably expressing *pSMAL* or *pSMAL-INPP4B* grown for 24 h as described above. Media was centrifuged at 200 x g and filtered through 0.45 µm filters to remove any cells. CM with or without immunodepletion was added to the upper chamber of 12-well ThinCert inserts and migrating cells assessed after 24h.

Collection and Immunodepletion of FN1 from CM

BxPC-3 cells were cultured at 5x10^5 cells per treatment condition per well of a 6-well plate for 24 hours in DMEM supplemented with 0.1% FBS. The CM was then collected and incubated with Protein A/G magnetic agarose beads (Thermo Fisher) for 2 hours with rotation to pre-clear. The beads were then removed using a DynaMag-2 Spin Magnet (Thermo Fisher). The pre-cleared media was incubated overnight with Protein A/G magnetic agarose beads conjugated to either a rabbit monoclonal anti-FN1 antibody (Cell Signalling Technologies, #26836) or normal rabbit IgG control (Cell Signalling Technologies, #2729), under continuous

rotation. Antibody-bound beads were separated from the media using the DynaMag-2 Spin Magnet. The collected CM were processed through two additional 24 h rounds of antibody incubation. The resulting FN1-depleted or IgG antibody treated media was either applied to BxPC-3 cells for transwell migration assays or heat-denatured for immunoblot analysis.

PDAC Patient Data analysis

FN1 and INP4B protein levels in PDAC patients were analyzed using UALCAN, based on data from the Clinical Proteome Tumour Analysis Consortium (CPTAC) (43). To examine *FN1* transcript expression in normal and PDAC pancreatic tissues, GEO datasets GSE15471 (44) and GSE16515 (45) were used. Additionally, GEPIA2 was employed to analyze data from The Cancer Genome Atlas-Pancreatic Ductal Adenocarcinoma study (TCGA-PAAD), assess correlations between *INPP4B* and *FN1* and *ITGB1* and *CDH1* levels, and perform Kaplan-Meier survival analyses (46).

Supplemental Figure legends

Supplementary Figure 1. Endogenous FN1 localises to lysosomes. *(A)* Representative micrographs of HPAC cells transfected with LAMP1-mCherry or Rab5-RFP constructs and immunostained for endogenous FN1 (green) and probed with DAPI. *(B)* Quantitation of FN1 intensity overlayed on LAMP1-mCherry or Rab5-RFP positive vesicles. *(C)* Representative micrographs of HPAC cells transfected with LAMP1-mCherry, treated with apilimod and immunostained for FN1 (green). Representative inset images illustrating individual LAMP1-mCherry positive vacuoles and FN1. *(D)* Percentage of LAMP1-mCherry vacuoles positive for luminal FN1. *(E)* HPAC cells transiently expressing LAMP1-mCherry, treated with vehicle or Bafilomycin and immunostained for FN1 (green). Image analysis of LAMP1 positive FN1 intensity *(F)* or whole cell FN1 intensity *(G)*. Scale bar: 5 µm *(A, E)* or 20 µm *(C)*. *(H)* Representative micrographs of BxPC-3 cells transduced with *pSMAL* or *pSMAL-INPP4B* transfected with LAMP1-mCherry and immunostained for FN1 (green) with DAPI. Scale bar: 10 µm. *(I, J)* Quantitation of Outer/Inner Shell FN1 intensity and Outer/Inner Shell LAMP1 intensity. Data represent ± SD from 90-100 cells assessed from three independent experiments per treatment condition.

Supplementary Figure 2. Wound healing of HPAC cells are INPP4B and FN1 dependent. (*A-C*) Quantitation and representative images of wound healing assays performed with *INPP4B* KO HPAC cells with and without 20 ug/mL FN1. Scale bar: 100 µm. Data represents at least three independent experiments per treatment condition.

Supplementary Figure 3. Evaluating FN1 expression from PDAC patients.

(A) FN1 protein expression in PDAC patients specimens and matched normal control from CPTAC data. *(B,C) FN1* transcript expression from normal and PDAC specimens. *(D,E)* Kaplan-Meier analysis of overall and disease free survival using TCGA-PAAD dataset

comparing high (top 25%) *FN1* expression compared to low (bottom 75%) *FN1* expression. *(F)* Correlation between *INPP4B* and *FN1* transcript expression from TCGA-PAAD. qRT-PCR was used to measure *FN1* in *INPP4B* KO HPAC *(G)* or BxPC-3 *(H)* cells or BxPC-3 cells stably expressing control *pSMAL* or *pSMAL-INPP4B (I).* Experimental data represent ± SD from three independent experiments per treatment condition.

Supplementary Figure 4. Effect of INPP4B expression E-cadherin and β1 integrin. Flow cytometry analysis of cell surface β1 Integrin *(A-C)* or cell surface E cadherin *(D-F)* for *INPP4B* KO HPAC *(A, D)* or *INPP4B* OE BxPC-3 *(B, E)* or *INPP4B* KO BxPC-3 cells *(C, F)*. Correlation between *INPP4B* and *ITGB1 (G)* or *CDH1 (H)* transcript expression from TCGA-PAAD. qRT-PCR of *CDH1 (I)* or *ITGB1 (J)* relative to *ACTB* in *INPP4B* KO HPAC or BxPC-3 cells or BxPC-3 cells stably expressing control *pSMAL* or *pSMAL-INPP4B.* Data represents ± SD from three independent experiments per treatment condition.
