# Supplementary figures and images for: The lipid phosphatase INPP4B controls pancreatic cancer cell migration and invasion by regulating fibronectin exocytosis
Source: J Biol Chem. 2025 Sep 15;301(10):110716. doi: 10.1016/j.jbc.2025.110716 (PMC12547244; doi:10.1016/j.jbc.2025.110716)

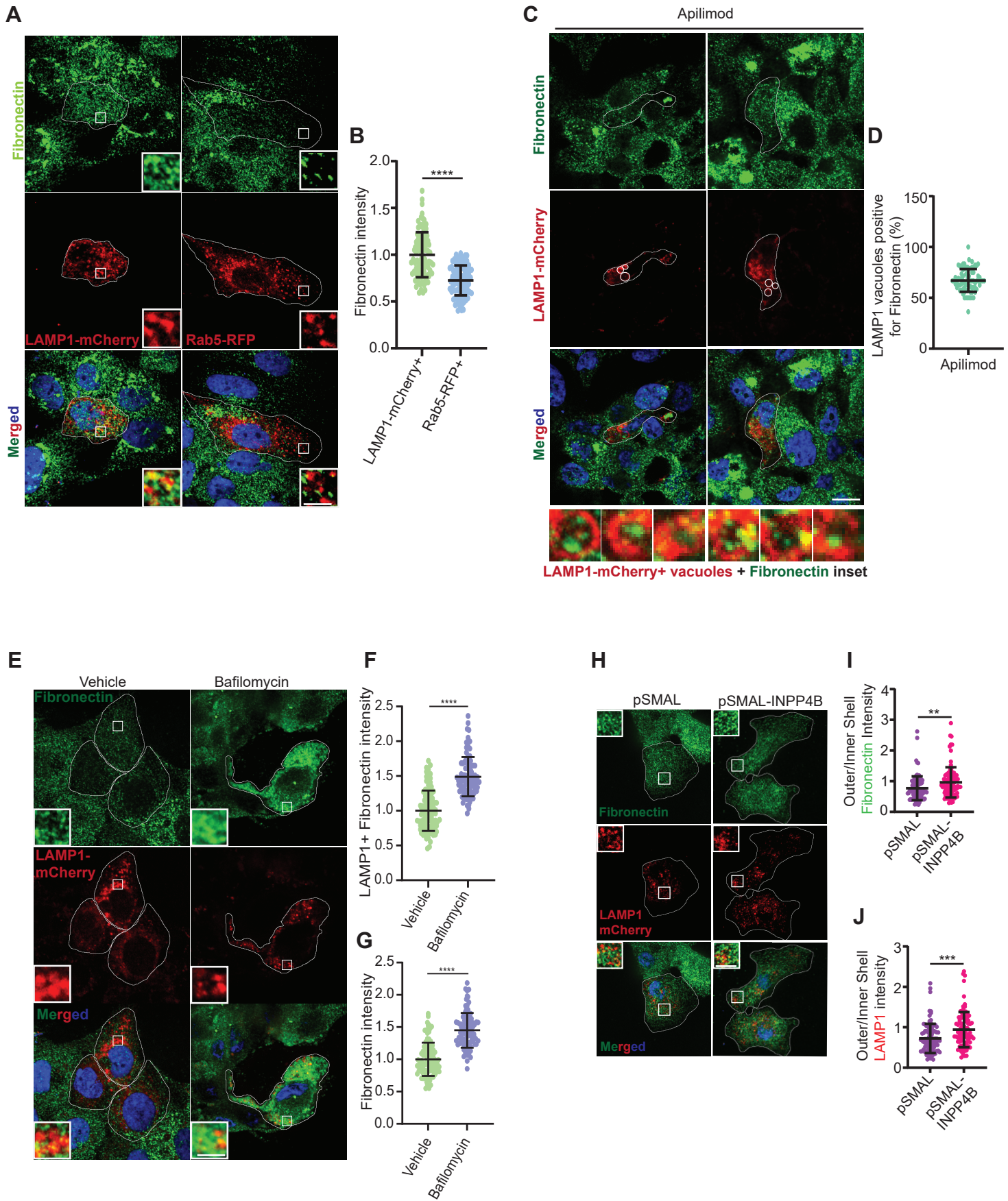

Supplement: Supplementary Figure 1 [file mmc2.pdf]

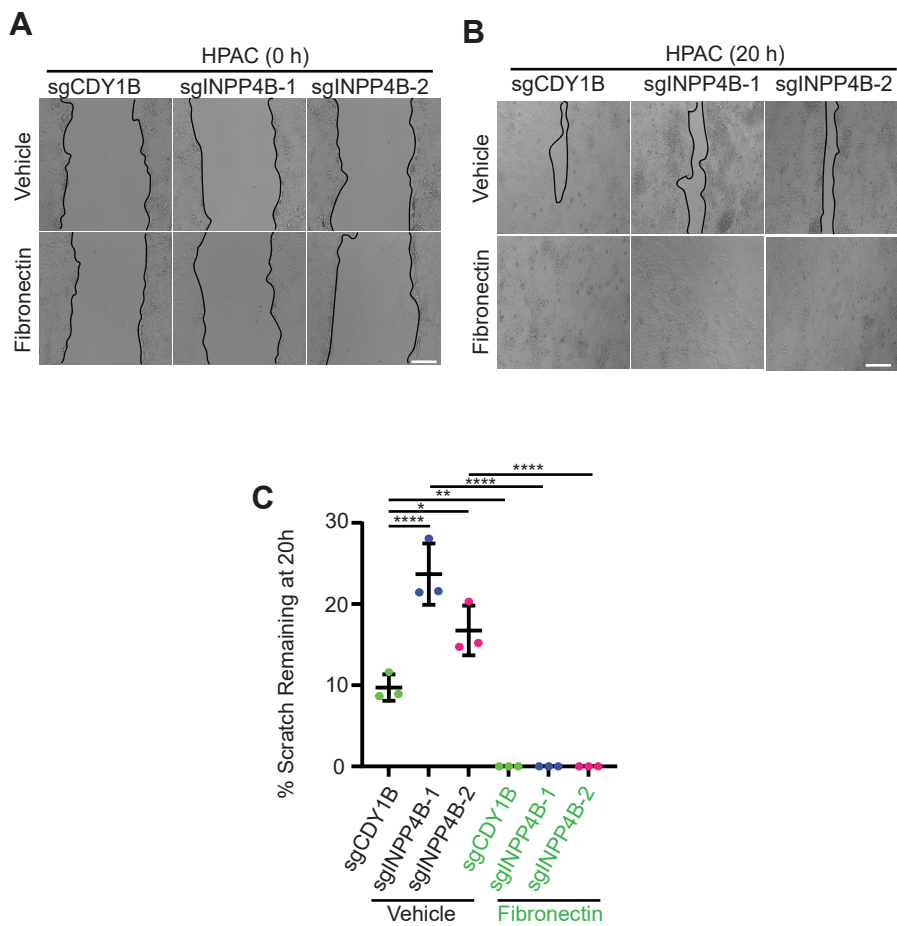

Supplement: Supplementary Figure 2 [file mmc3.pdf]

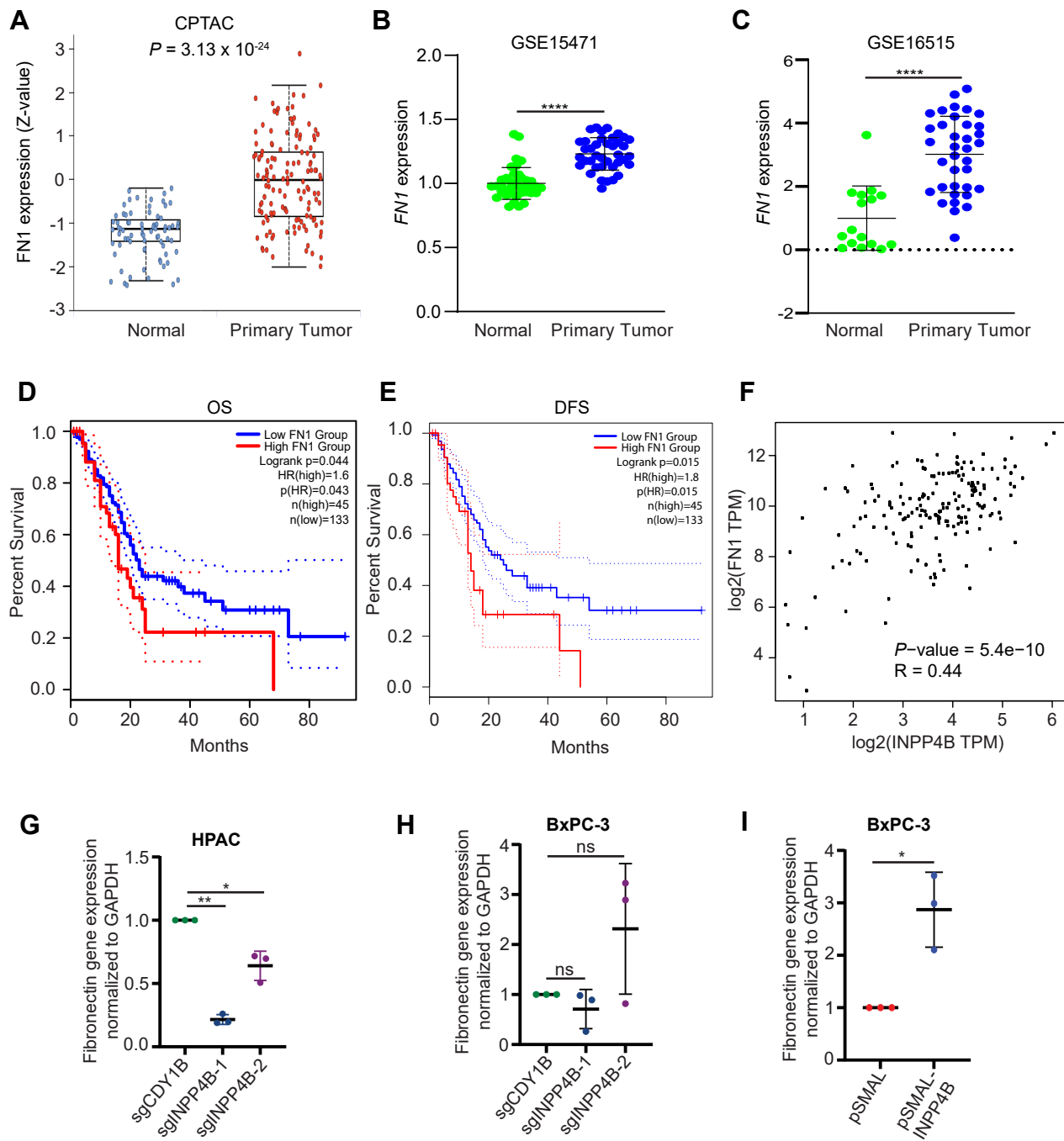

Supplement: Supplementary Figure 3 [file mmc4.pdf]

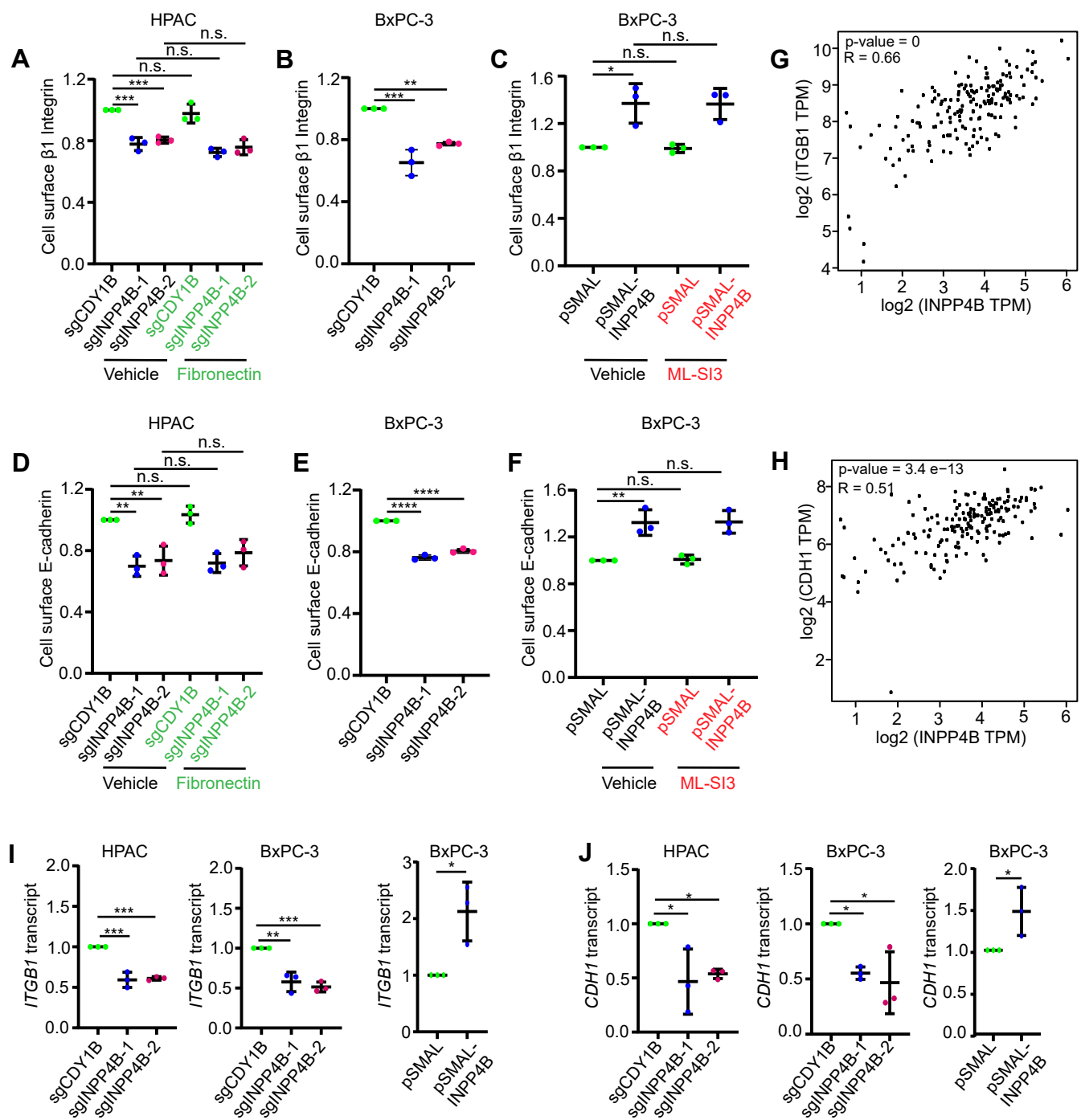

Supplement: Supplementary Figure 4 [file mmc5.pdf]
