# Supplementary material for: The lipid phosphatase INPP4B controls pancreatic cancer cell migration and invasion by regulating fibronectin exocytosis
Source: J Biol Chem. 2025 Sep 15;301(10):110716. doi: 10.1016/j.jbc.2025.110716 (PMC12547244; doi:10.1016/j.jbc.2025.110716)

Figure 1A HPAC INPP4B (110 kDA)

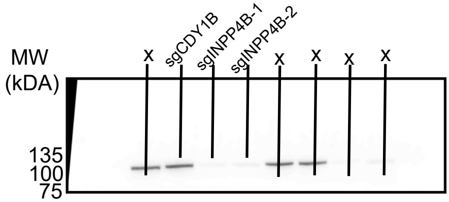

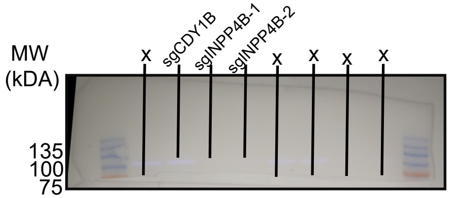

Figure 1A HPAC Actin (45 kDA)

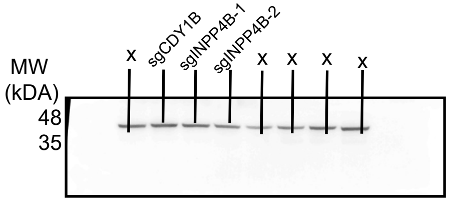

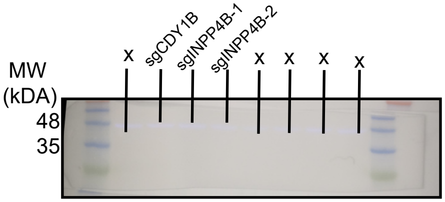

Figure 1F BxPC-3 INPP4B (110 kDA)

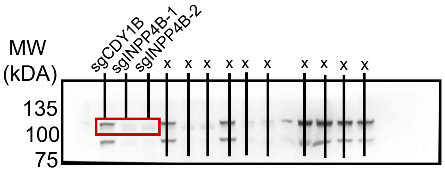

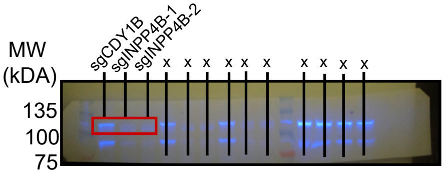

Figure 1F BxPC-3 Actin (45 kDA)

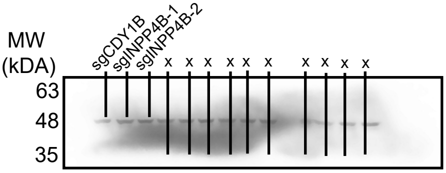

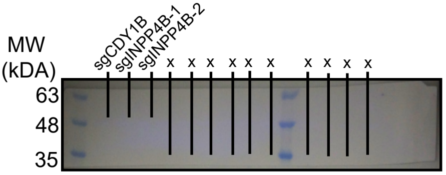

Figure 1I BxPC-3 INPP4B (110 kDA)

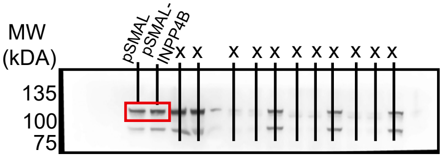

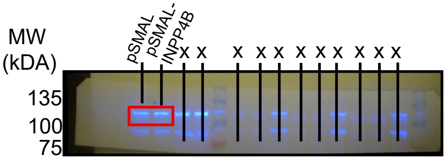

Figure 1I BxPC-3 Actin (45 kDA)

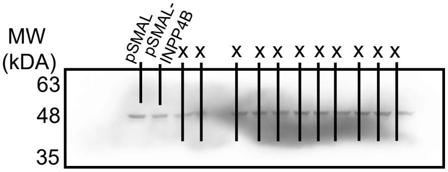

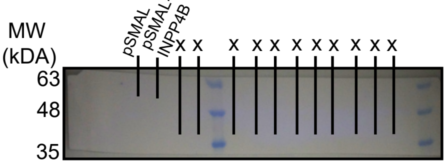

Figure 4G Fibronectin (250 kDA)

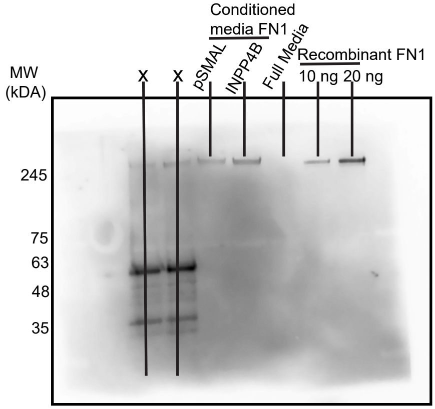

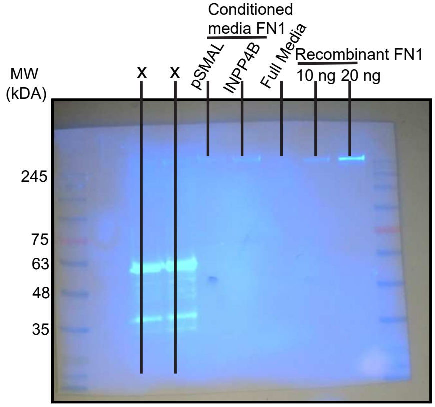

Figure 5G Fibronectin (250 kDA)

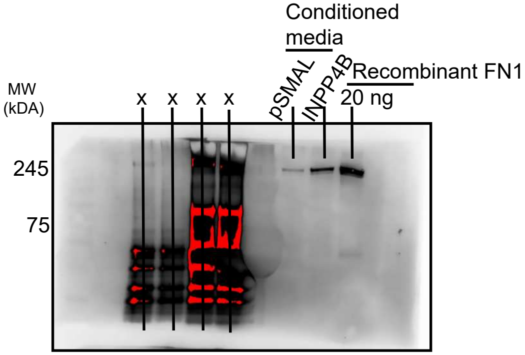

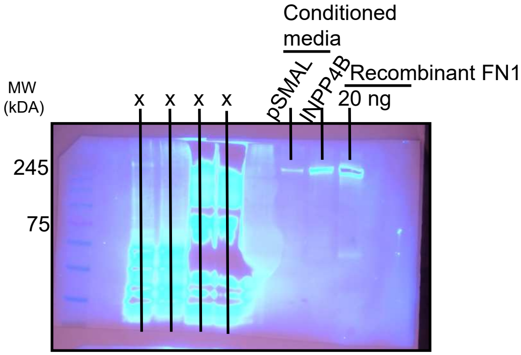

Figure 5I Fibronectin (250 kDA)

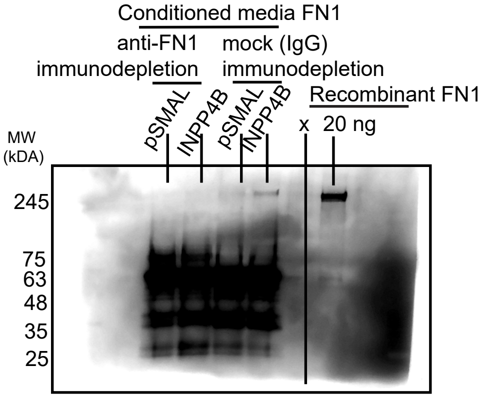

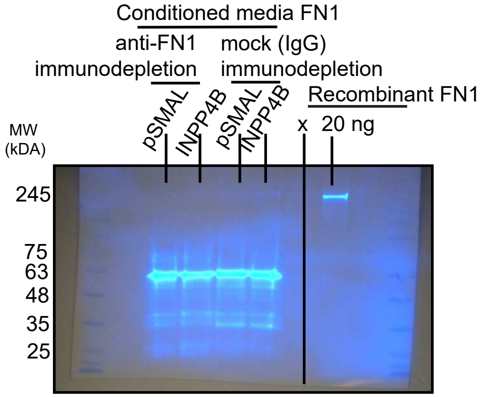

Supplement: Supplemental Figure Raw Western Blot images [file mmc6.pdf]
